# BuZhong YiQi Formula Alleviates Taste Disorders in Rats with Type 2 Diabetes Mellitus by Increasing the Number of Taste Buds and the Expression of Signaling Molecules in Taste Transduction Pathways

**DOI:** 10.3390/ph18060838

**Published:** 2025-06-03

**Authors:** Zhen-Ran Hu, Xiang-Ke Li, Guo-Jun Fei, Ming-Yu Wang, Meng-Juan Luo, Xin-Xin Zeng, Liang Wang, Ze-Min Yang

**Affiliations:** 1 Department of Biochemistry and Molecular Biology, School of Basic Medical Sciences, Guangdong Pharmaceutical University, Guangzhou 510006, China; huzhenran1217@163.com (Z.-R.H.); kekeli123123@163.com (X.-K.L.); feiguojun2024@163.com (G.-J.F.); wmy112222@163.com (M.-Y.W.); lmj8385936247@163.com (M.-J.L.); zengxinxin27@163.com (X.-X.Z.); liang7982023@163.com (L.W.); 2 Guangdong Provincial Key Laboratory of Pharmaceutical Bioactive Substances, Guangdong Pharmaceutical University, Guangzhou 510006, China

**Keywords:** type 2 diabetes mellitus, buzhong yiqi formula, taste disorders, sweet taste receptors pathway, taste bud organoids

## Abstract

**Background:** Taste disorders in patients with type 2 diabetes mellitus (T2DM) have a negative impact on their quality of life and glycemic control, and treatment options are limited. Buzhong yiqi formula (BZYQF) improves T2DM symptoms but its effects on T2DM-induced taste disorders have not been sufficiently studied. **Methods**: Molecular docking was utilized to evaluate binding activity between the compounds in BZYQF and the sweet taste receptors (STRs). T2DM was induced in rats through the administration of high-fat diet and streptozotocin, and the rats were then treated with BZYQF for 8 weeks. Daily indicators and serum biochemical factors were monitored. Taste preferences for sweet, bitter, salty, and sour solutions were assessed using a two-bottle test. The morphology of lingual papillae and the numbers of taste buds were examined using HE staining. A high-glucose (HG) model of taste bud organoids was established to measure sucrose-evoked ATP release. The expression of signaling molecules in the sweet taste receptors (STRs) pathway was determined via RT-qPCR, Western blot, and immunofluorescence in lingual papillae and organoids. **Results**: A total of 508 compounds in BZYQF indicated good binding activity to T1R2, T1R3 or heterodimers of T1R2/T1R3, and 60 compounds had good binding activity to all three forms of STRs. BZYQF alleviated T2DM symptoms and improved taste perception for maltose (10 mM, 50 mM), quinine (0.03 mM, 0.1 mM), and citric acid (1 mM) solutions. BZYQF improved the morphological structure of lingual papillae and increased taste bud numbers in T2DM rats. BZYQF enhanced ATP release responses to sucrose solution in the taste bud organoids of the HG model. Gene expression determination showed that BZYQF upregulated the expression of signaling molecules in the STRs pathway (T1R2, T1R3, IP3R, α-gustducin, TRPM5) in the lingual papillae of the T2DM rats and in the taste bud organoids of the HG model. **Conclusions**: BZYQF alleviates T2DM-induced taste disorders by increasing the numbers of taste buds and upregulating STR signaling molecules, in which various compounds, especially flavonoids, exhibit a synergistic effect.

## 1. Introduction

T2DM is a chronic endocrine metabolic disease characterized by hyperglycemia. This disease commonly occurs in middle-aged and older adults, as well as in obese individuals, and is often accompanied by complications of various organs. Oral health problems are usually associated with T2DM and manifest as dry mouth, periodontitis, and salivary gland dysfunction [1]. T2DM can also affect taste perception. Several studies have suggested that individuals with T2DM generally experience impaired taste perception, particularly the worsening of sweet taste perception, compared with healthy individuals [2,3]. Taste perception plays an important role in determining food preferences and shaping individual dietary habits, which may impact the progression of T2DM [4]. For example, individuals with impaired taste perception for sweet and salty foods may consume more sugar and sodium to achieve the same taste satisfaction, ultimately leading to rapid increases in blood glucose and blood pressure levels and affecting the management of blood glucose stability [5,6]. Therefore, improving T2DM-induced taste disorders is crucial for T2DM patients to maintain reasonable nutritional intake and stable glycemic control, as well as to improve their quality of life.

The tongue is considered the primary taste sensory organ, sensing taste through papillae on its surface. There are four types of papillae: filiform, fungiform (FPs), foliate, and circumvallate (CVPs). All papillae except for the filiform papillae contain taste buds. Each taste bud consists of 50–100 chemosensory cells derived from the epithelium, which are known as taste cells. These taste cells can be divided into three types, i.e., I, II, and III. Type I cells provide structural support and may play a role in salty taste detection. Type II cells express G protein-coupled receptors (GPCRs), which are responsible for detecting sweetness, bitterness, and umami. Among the GPCRs, the type 1 taste receptor family (T1R) detects sweetness (T1R2/T1R3 heterodimers) and umami (T1R1/T1R3 heterodimers), while the type 2 taste receptor family (T2R) with 25–30 subtypes is responsible for the recognition of various bitter tastes. The detection of sweet, bitter, and umami elements is mediated by similar taste signaling pathways. These taste elements such as sweet, bitter, and umami bind to GPCRs, activating α-gustducin and opening the transient receptor potential cation channel subfamily M member 5 (TRPM5) via a canonical phospholipase C-β2 (PLCβ2)/inositol trisphosphate (IP3) signaling cascade, finally releasing ATP through the CALHM1 channel and delivering the taste signal via the sensory afferent fiber. Type III cells may be involved in sour taste recognition [7]. The taste sensory system is responsible for examining food nutrition and avoiding ingestion of harmful substances. However, the relationship between taste perception and T2DM is unclear and requires further clarification.

At present, the treatment of T2DM mainly relies on chemical drugs, which have significant effects on controlling hyperglycemia in T2DM patients. However, these chemical drugs have little effect on alleviating the complications in T2DM patients, especially taste disorders, and they may also be associated with significant side effects such as liver and kidney toxicity and gastrointestinal problems [8]. In contrast, Chinese herbs improve blood glucose levels in T2DM patients with minimal side effects and reduce the risk of T2DM complications through the synergistic effects of multiple components, multiple targets and multiple pathways [9]. Buzhong yiqi formula (BZYQF) is a classic formula in traditional Chinese medicine. The clinical applications of BZYQF are primarily in the treatment of spleen-Qi deficiency [10] and show remarkable effectiveness in improving digestive and immune functions [11]. Clinical studies have shown that BZYQF can reduce hyperglycemia and improve insulin sensitivity in T2DM patients [12]. Nevertheless, the treatment of T2DM-induced taste disorders with BZYQF has not been reported, and the mechanism involved of BZYQF in treating T2DM needs to be elucidated.

The current research on taste disorders in T2DM mainly relies on animal models and clinical investigations. However, due to the limitations in obtaining sufficient taste bud cells from humans and animals for in vitro studies, the underlying molecular mechanisms of T2DM-induced taste disorders remain unclear. The advent of organoid technology has overcome this limitation, enabling the long-term cultivation of taste bud cells from lingual papillae and opening new opportunities for conducting mechanistic studies in vitro. As a three-dimensional model of stem cell culture in vitro, taste bud organoids closely resemble taste buds in vivo. These organoids have been confirmed to express mature taste markers and responses to chemical taste stimulation [13,14]. However, few studies report findings relating to taste disorders induced by T2DM using taste bud organoids.

In this study, we first analyzed the affinity of compounds in BZYQF to sweet taste receptors (STRs) of T1R2/T1R3 and assessed the therapeutic potential of BZYQF on metabolic disorders and taste disorders in T2DM rats. We then examined the morphological structure of the lingual papillae and the numbers of taste buds they contained, as well as the gene expression of signaling molecules in the STR signaling pathway. Finally, a taste bud organoid model was developed to evaluate the influence of high glucose and BZYQF on the taste bud organoids’ response to sucrose stimulation and on the gene expression of STR signaling molecules. This study’s results provide a theoretical basis for the use of BZYQF in improving taste disorders caused by T2DM.

## 2. Results

### 2.1. Prediction and Affinity Analysis of Compounds Regulating STRs in BZYQF

**Figure 1:**
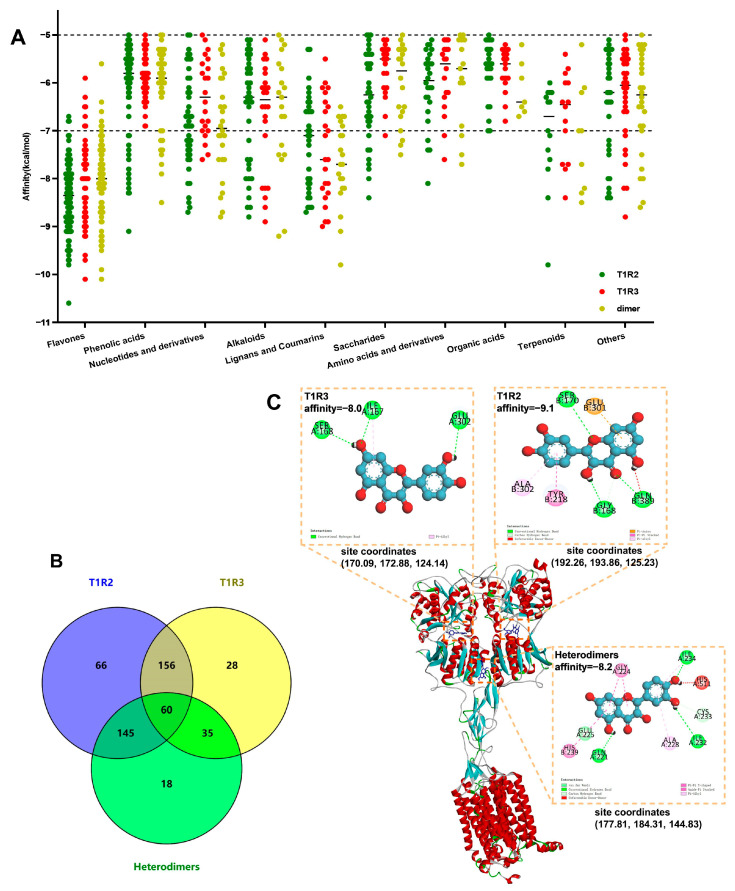
Molecular docking between the identified compounds in BZYQF and heterodimers of T1R2/T1R3. (**A**) The affinity of different compounds in BZYQF with T1R2, T1R3, and heterodimers. (**B**) Venn diagram of compounds with good binding activity to T1R2, T1R3, or heterodimers. (**C**) The docked 3D and 2D diagrams of compounds in BZYQF to T1R2, T1R3, and heterodimers. Quercetin was shown to be one of the 60 compounds which had a good binding activity to all three forms of T1R2, T1R3, and heterodimers.

To investigate the compounds in BZYQF that can regulate STRs, molecular docking was conducted to examine the interaction between the compounds identified via UPLC-MS/MS and heterodimers of T1R2/T1R3. Out of the 1909 identified compounds in BZYQF, 2D structural information for 682 compounds was found in the PubChem database. Furthermore, molecular docking indicated that a total of 427, 279, and 258 compounds exhibited good binding activity to T1R2, T1R3, and heterodimers of T1R2/T1R3 (affinity < −5.0 Kcal/mol), respectively (Figure 1B). These compounds were mainly flavonoids and phenolic acids, which accounted for 41.1% of the compounds docked with T1R2, 39.3.1% of the compounds docked with T1R3, and 43.7% of the compounds docked with heterodimers, respectively (Figure 1A). The affinity values indicated that compared with other compounds, flavonoids, lignans, and coumarins had stronger binding activity with T1R2, T1R3, and heterodimers, demonstrating strong binding activity (average affinity < −7.0 Kcal/mol, Figure 1A). These compounds with binding activity were docked to the T1R2 sites at x 191.71 ± 3.51, y 193.58 ± 5.06, z 126.37 ± 12.25, docked to the T1R3 sites at x 169.47 ± 7.76, y 176.18 ± 10.51, z 123.67 ± 7.53, and docked to the heterodimer sites at x 179.64 ± 4.50, y 182.28 ± 5.19, z 139.58 ± 17.27 (Figure 1C). Among the identified compounds of BZYQF, 60 compounds had a good binding activity to all three forms of T1R2, T1R3, and heterodimers (Figure 1B). The statistical results regarding the amino acid binding sites showed that when docked to the binding sites of T1R2, the 60 compounds of BZYQF were mainly surrounded by 11 amino acid residues: ALA 302, TYR 218, GLU 301, GLY 168, GLN 193, GLN 326, ASP 190, SER 170, PRO 188, GLN 389, TYR 454, etc. When docked to the binding sites of T1R3, these compounds were mainly surrounded by 13 amino acid residues: TYR 215, GLU 302, SER 144, ILE 167, TYR 103, PRO 277, LYS 65, SER 168, SER 303, ASP 278, ASP 142, SER 165, GLU 145, etc. When docked to the heterodimer binding sites, these compounds were mainly surrounded by 11 amino acid residues: HIS 239 and GLN 262 of T1R2, and HIS 511, ALA 228, ILE 234, GLN 221, GLY 224, ARG 256, GLU 225, CYS 233 and ILE 510 of T1R3, etc. These results revealed that most of the compounds in BZYQF had good binding activity with the three forms of STRs, and their binding sites with the same form of STRs were consistent, suggesting that they might regulate STRs through synergistic effects and improve taste disorders.

### 2.2. BZYQF Improved the Symptoms and Metabolic Levels in T2DM Rats

**Figure 2:**
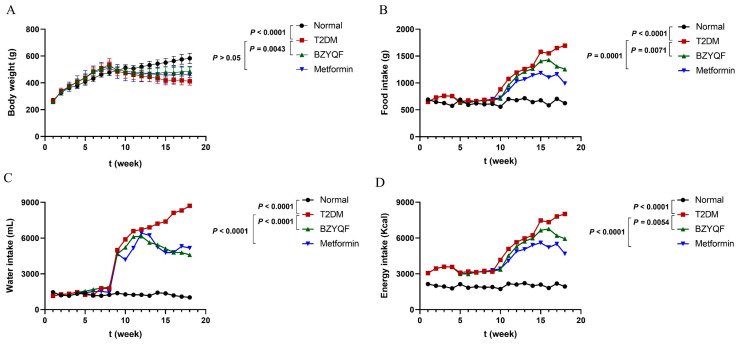
Daily indicators in experimental rats. T2DM rats had higher intakes of (**B**) food, (**C**) water, and (**D**) energy, and lower (**A**) body weight than normal rats. After drug treatment for 8 weeks, BZYQF significantly decreased intakes of water, food, and energy, and increased body weight in T2DM rats. There were 8 rats in each group, except for 6 rats in the metformin group.

In order to evaluate the therapeutic effect of BZYQF on T2DM rats, this study observed the daily and biochemical indicators of experimental rats. The results of the daily indicators are shown in Figure 2. After a single STZ injection (week 10), the T2DM rats showed an obvious increase in the intakes of water, food and energy, and a remarkable decrease in body weight compared with normal rats. The T2DM rats exhibited typical symptoms of polyuria (the padding in T2DM rats was wetter than in normal rats), polyphagia, polydipsia, and weight loss. Over the following 8 weeks, these daily indicators of the T2DM rats became more typical. After drug treatment for 8 weeks (week 18), the BZYQF-treated rats demonstrated significant improvements in water intake, food intake, energy intake, and body weight compared with the T2DM rats, and similar results were found in the metformin-treated rats, except for body weight. The results for the biochemical indicators observed in serum at the end of the experiment (week 18) are shown in Table 1. The T2DM rats had higher levels of FBG, total cholesterol (TC), and triglyceride (TG), and lower insulin levels and HOMA-β than the normal rats. After drug treatment for 8 weeks, all of these indicators were markedly improved the in BZYQF-treated rats compared with those in T2DM rats, including FBG (*p* = 0.0058), TC (*p* < 0.0001), TG (*p* < 0.0001), insulin (*p* = 0.0017), and HOMA-β (*p* = 0.0106), and similar results were found in the metformin-treated rats. These findings indicate that BZYQF had a significant therapeutic effect on T2DM rats, manifested as improvements in symptoms and glucose and lipid metabolism.

### 2.3. BZYQF Improved Taste Disorders in T2DM Rats

**Figure 3:**
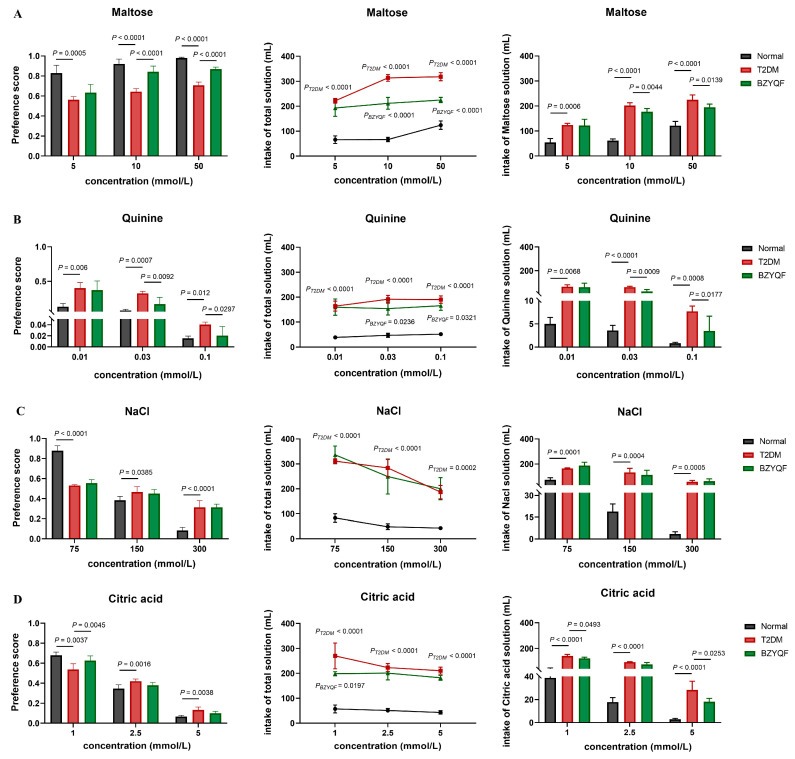
Preference values and intakes of solution in experimental rats in a two-bottle preference test. T2DM rats had abnormal preference scores and intakes of four solutions of (**A**) maltose, (**B**) quinine, (**C**) NaCl, and (**D**) citric acid, while BZYQF improved preference scores and intakes of maltose and quinine solutions at medium and high concentrations, and citric acid solution at low concentration. *P_T2DM_*: compared with normal rats. *P_BZYQF_*: compared with T2DM rats. The number of rats in each group was 8.

To examine the taste disorders caused by T2DM, a two-bottle preference test was used to detect the responses of the experimental rats to different tastant solutions. The results are shown in Figure 3. In the sweet taste test (Figure 3A), the normal rats showed a clear preference for maltose (preference score > 0.5), and their preference score increased with the increasing concentration of the maltose solution. The T2DM rats showed a weak preference for maltose solutions at all three concentrations, and their preference scores were significantly lower than those of the normal rats. After drug treatment, the BZYQF-treated rats showed a clear preference for maltose solutions at concentrations of 10 mM (*p* < 0.0001) and 50 mM (*p* < 0.0001), with preference scores markedly higher than those of the untreated T2DM rats; however, the treatment had no effect on the preference score for 5 mM maltose in the T2DM rats. Moreover, the T2DM rats had higher intakes of total solutions and maltose solutions at three different concentrations than the normal rats. BZYQF significantly decreased the intakes of total solution and maltose solution at 10 mM (P_total_ < 0.0001, P_maltose_ = 0.0044) and 50 mM (P_total_ < 0.0001, P_maltose_ = 0.0139), but it had no effect on the intakes of total solution and maltose solution at 5 mM in the T2DM rats. These results suggest that the T2DM rats exhibited reduced perceptions of sweet taste, and BZYQF treatment significantly improved their perception for sweet solutions at medium and high concentrations.

In the bitter taste test (Figure 3B), all rats showed an obvious aversion to quinine (preference score < 0.5), and their preference scores decreased as the concentration of quinine solution increased. The T2DM rats had significantly higher preference scores for quinine solutions at three different concentrations than the normal rats. After drug treatment, the preference scores for quinine solutions at concentrations of 0.03 mM (*p* = 0.0092) and 0.1 mM (*p* = 0.0297) were observed to decrease in the BZYQF-treated rats compared with those for the T2DM rats, while there was no difference in preference score for the quinine solution at 0.01 mM between the two groups of rats. Additionally, the T2DM rats had higher intakes of total solutions and quinine solutions at three different concentrations than the normal rats. Conversely, BZYQF treatment significantly reduced the intakes of total solution and quinine solutions at 0.03 mM (P_total_ = 0.0236, P_quinine_ = 0.0009) and 0.1 mM (P_total_ = 0.0321, P_quinine_ = 0.0177) in the T2DM rats, but had no effect on the intakes of total solution and quinine solution at 0.01 mM. These results suggest that the T2DM rats showed a reduced perception of bitter taste and that BZYQF restored this impairment at medium and high concentrations.

In the salt taste test (Figure 3C), all rats showed a preference for the NaCl solution at a concentration of 75 mM and an apparent aversion to those of 150 mM and 300 mM, with preference scores decreasing as the concentration of NaCl increased. The T2DM rats had significantly lower preference scores for the NaCl solutions at 75 mM and higher scores for those at 150 mM and 300 mM than the normal rats. After drug treatment, there were no differences in the preference scores for any of the NaCl solution concentrations between the BZYQF-treated and T2DM rats. Additionally, the T2DM rats consumed more total solution and NaCl solutions at all three concentrations than the normal rats. However, no significant differences were observed in the intake of total solution or the NaCl solutions at the three concentrations between the BZYQF-treated rats and the T2DM rats. These results showed that BZYQF treatment did not improve the T2DM rats’ reduced perception of saline flavor.

In the sour taste test (Figure 3D), all rats showed a preference for the citric acid solution at 1 mM and an apparent aversion to those at higher concentrations (2.5 mM and 5 mM). Their preference scores decreased with increasing concentrations of citric acid solution. Compared with the normal rats, the T2DM rats had significantly lower preference scores for the citric acid solution at a concentration of 1 mM, but higher scores for those at 2.5 mM and 5 mM. After drug treatment, the BZYQF-treated rats showed a clear preference for citric acid solutions at 1 mM (*p* = 0.0450) with scores significantly higher than those of the untreated T2DM rats. However, BZYQF treatment had no effect on the preference scores for the 2.5 mM and 5 mM solutions in T2DM rats. Moreover, the T2DM rats had higher intakes of total solutions and citric acid solutions at all three different concentrations than normal rats. BZYQF treatment significantly decreased the intakes of total solution and citric acid solution at 1 mM (P_total_ = 0.0179, P_citric acid_ = 0.0493). BZYQF also reduced the intake of the citric acid solution at 5 mM (P_citric acid_ = 0.0253), but not total solution. However, the treatment had no effect on the intakes of total solution or the citric acid solution at 2.5 mM in the T2DM rats. These results suggest that the T2DM rats had an impaired perception of sour taste, and BZYQF treatment reverted this impairment at low concentrations.

These experiments showed that the T2DM rats exhibited impairments in taste characterized by reduced perception of sweet, bitter, salty, and sour flavors, as evidenced by the higher intakes of total solution and tastant solution. Treatment with BZYQF was found to clearly reverse T2DM-induced taste disorders, except for salty solutions.

### 2.4. BZYQF Improved the Morphological Structure and Number of Taste Buds in FPs and CVPs in T2DM Rats

**Figure 4:**
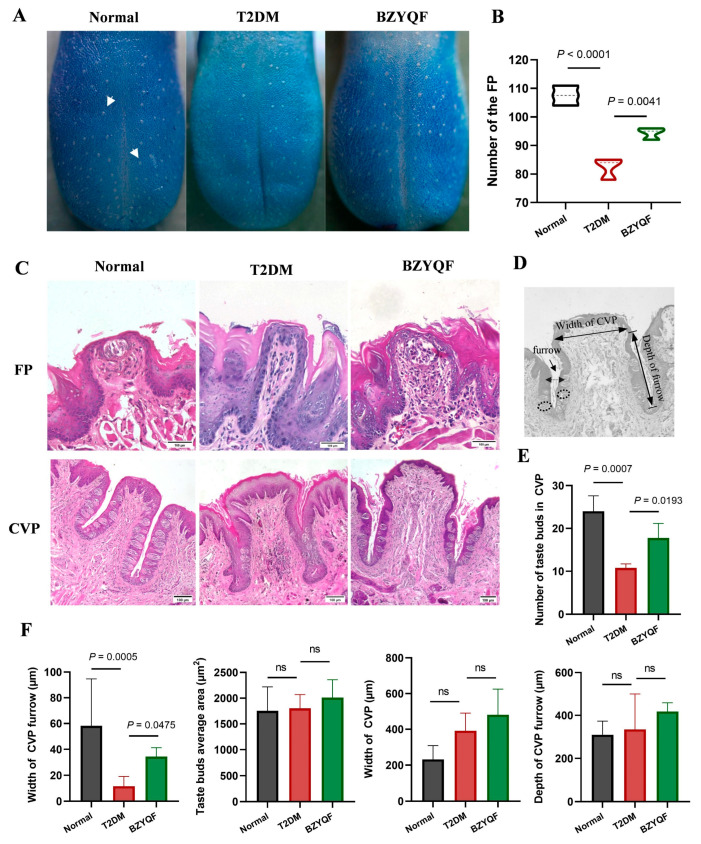
Morphological structure and dimensions of the lingual papillae. (**A**,**B**) Methylene blue staining and counting for FP. The white arrows indicated the position of the FP. (**C**) HE staining of paraffin-embedded tongue slices from the experimental rats. (**D**–**F**) Morphological dimensions of CVP. BZYQF significantly increased the number of taste buds and improved the morphological structure of FP and CVP in T2DM rats. ns: not significant. Scale bar = 100 μm. The number of rats in each group was 4.

To investigate the histological mechanisms underlying these T2DM-induced taste disorders and the beneficial effects of BZYQF, we observed the morphological structures of FPs and CVPs and the numbers of taste buds in the experimental rats. As shown in Figure 4A,B, the numbers of FPs on the tongue surface were lower in the T2DM rats than that in normal rats. After drug treatment, BZYQF significantly increased the numbers of FPs in the T2DM rats. The results from the HE staining of FPs are illustrated in Figure 4C-FP. There was a single intact taste bud in every FP of the normal rats, but many were absent from the FPs of T2DM rats. However, in the BZYQF-treated rats, these single taste buds could be observed in FPs, although the structure of these taste buds was loose.

The results from the HE staining of CVPs are shown in Figure 4C–F. In the normal rats, the morphological structure of the taste buds was regular and neatly arranged, with clearly visible furrows and a tightly connected stratum corneum. However, in the T2DM rats, the morphological structure of the taste buds was irregular and disorderly, with blurred furrows and a loose stratum corneum. After drug treatment, BZYQF significantly improved these histopathological structures in the T2DM rats. The dimensional assessments of the CVPs revealed significant reductions in taste bud numbers and furrow width in the T2DM rats compared with those in normal rats. After drug treatment, BZYQF significantly increased the numbers of taste buds and the furrow width in the T2DM rats. However, there were no differences in these indicators, including the width of the CVP, taste bud average area, and furrow depth in the CVP, among the three groups of rats. These results indicated that there were histopathological changes in the lingual papillae of the T2DM rats, and that BZYQF treatment increased the number of taste buds and improved the morphological structure of FPs and CVPs.

### 2.5. BZYQF Upregulated the mRNA and Protein Expressions of STR Signaling Molecules in FP and CVP of T2DM Rats

**Figure 5:**
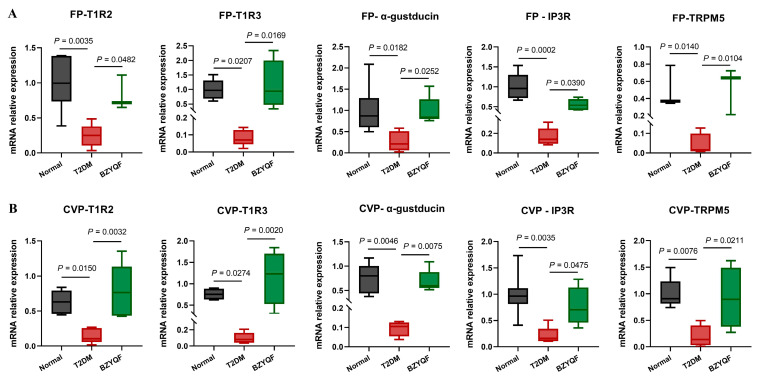
The mRNA expressions of STR signaling molecules in the FP (**A**) and CVP (**B**). T2DM rats had a reduced mRNA expression of STR signaling molecules in the FP and CVP, while BZYQF increased significantly the mRNA expressions of T1R2, T1R3, α-gustducin, IP3R and TRPM5 in the FP and CVP of T2DM rats. The number of rats in each group was 4.

In order to explore the molecular mechanisms underlying T2DM-induced taste disorders and the beneficial effects of BZYQF, we detected the expressions of STR signaling molecules in the FPs and CVPs. As shown in Figure 5, compared with the normal rats, the T2DM rats exhibited a significant reduction in mRNA expression of the STR signaling molecules, including T1R2, T1R3, α-gustducin, IP3R, and TRPM5, in FPs and CVPs. After drug treatment, the mRNA expressions of these STR signaling molecules in the FPs and CVPs significantly increased in the BZYQF-treated rats compared with those in the T2DM rats. Furthermore, no significant difference in the mRNA expressions of these STR signaling molecules was found between FPs and CVPs.

**Figure 6:**
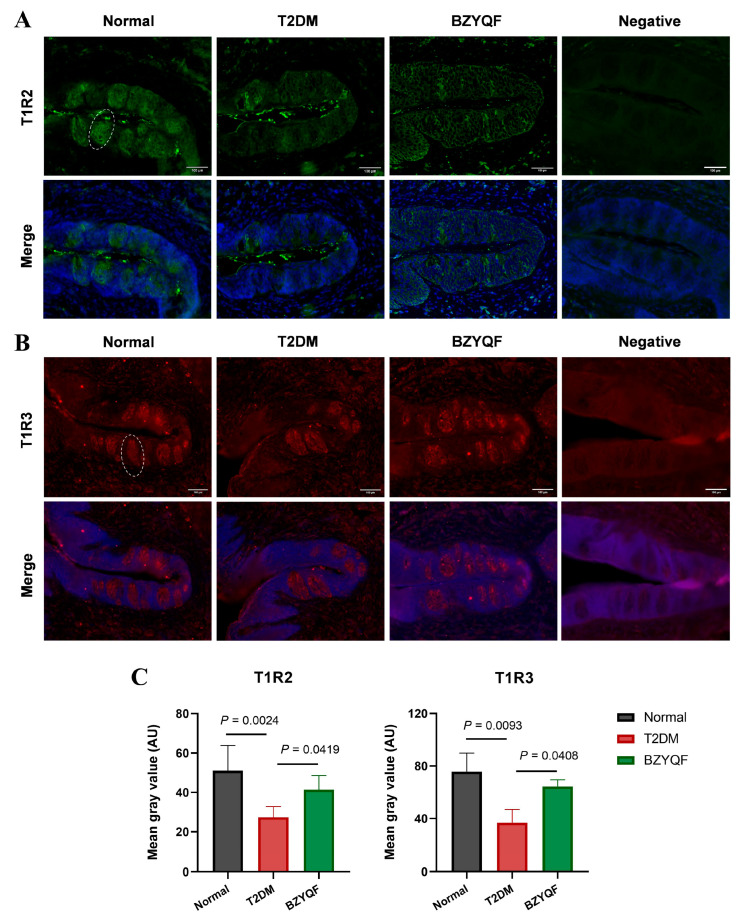
Immunohistochemical staining for T1R2 (green) and T1R3 (red) in the CVP. (**A**,**B**) Immunoreactivities for T1R2 and T1R3 were observed in the taste buds (white dashed circles). The nuclei were counterstained with DAPI (blue). The merged images were displayed in the lower panels. Negative: No immunoreactivity was observed when primary antibodies were omitted. (**C**) Morphometric analysis of T1R2 and T1R3 staining intensity. Mean gray value = IntDen/area; AU, arbitrary unit. Scale bar = 100 μm. The number of rats in each group was 4.

The localization of T1R2 and T1R3 immunoreactivity in the taste buds of the CVPs is shown in Figure 6. The taste buds expressing T1R2 and T1R3 exhibited intact and recognizable morphology in the normal rats, while the structure of the taste buds appeared blurred in the T2DM rats. The immunofluorescence intensities of T1R2 and T1R3 in CVPs were lower in the T2DM rats than in normal rats, and a similar result was found in the mean gray value according to semi-quantification analysis. After drug treatment, BZYQF significantly improved the taste bud morphology and increased the fluorescence intensity of T1R2 and T1R3 in T2DM rats. These results revealed that the expressions of STR signaling molecules in the FPs and CVPs of T2DM rats were impaired, and that BZYQF restored the expressions of these signaling molecules in T2DM rats.

### 2.6. Morphological and Functional Properties of Taste Bud Organoids

**Figure 7:**
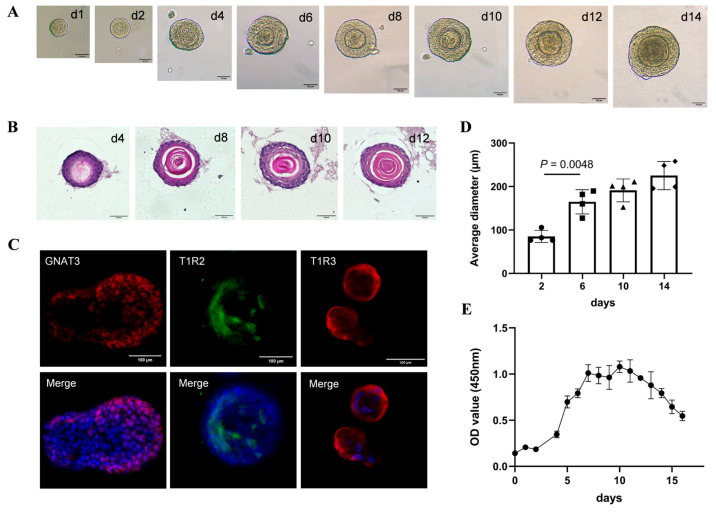
Growth and functional characterization of taste bud organoids from the CVP. (**A**) Pho-tograph of taste bud organoids on different culture days. (**B**) HE staining for organoids on different culture days. (**C**) Immunofluorescence staining with GNAT3 (red), T1R2 (green), T1R3 (red), and cell nuclei (blue) for organoids. (**D**) The average diameter of organoid colonies on day 2, 6, 10, and 14, respectively. (**E**) The growth curves of organoids based on the absorbance values of CCK-8 assay. ns: not significant. Scale bar = 100 μm.

To assess the feasibility of organoids as a model for T2DM-induced taste disorders, we performed morphological observations and functional validation for taste bud organoids. First, the growth of the organoids was monitored through photography. By day 6, the average diameter of the organoids had increased from 100 μm to 400 μm (Figure 7D), which ultimately led to the formation of round organoids with a concentric cell arrangement, and the organoids subsequently continued to grow larger (Figure 7A). Daily proliferation assays of organoids revealed that there was a rapid growth phase from day 4 to day 7, followed by a stable phase from day 7 to day 12 and a degradation phase from day 12 to day 16 (Figure 7E). Histological examination with HE staining showed a stratum corneum stained red with the eosin reagent in organoids with concentric cell arrangements (Figure 7B), and the staining intensity increased with culture time. Immunofluorescence staining confirmed that GNAT3 (red), T1R2 (green), and T1R3 (red), markers of type II taste cells, were expressed on the taste bud cells (Figure 7C). These results suggest that CVP-derived stem cells may generate mature taste bud organoids in vitro and have cellular properties similar to those of native taste buds, which could serve as an in vitro model for T2DM-induced taste disorders for drug development.

### 2.7. BZYQF Increased ATP Release Responses to Sucrose Solution in the High-Glucose Model of Taste Bud Organoids

**Figure 8:**
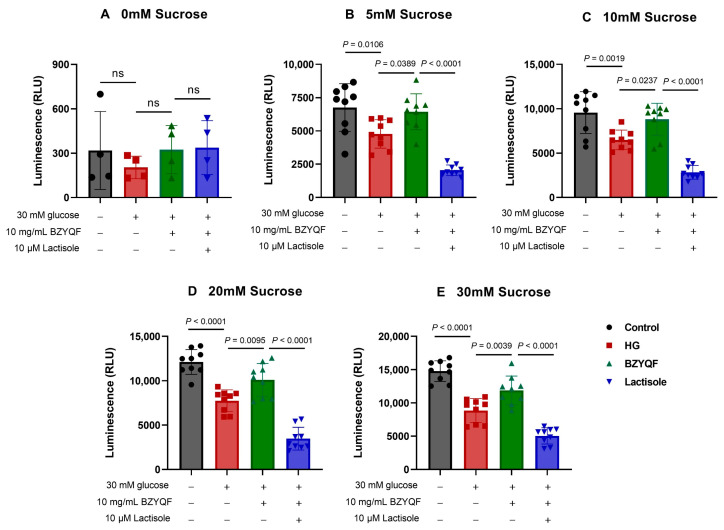
ATP release responses to sucrose solutions at different concentrations in taste bud organoids. The glucose of high concentration reduced the ATP release responses to sucrose solutions of different concentrations in taste bud organoids, while BZYQF reversed the ATP release responses to sucrose solutions in organoids of the HG group. However, this effect of BZYQF could be blocked by the T1R3 antagonist lactisole. ns, not significant.

To further investigate the influence of glucose of high concentration on sweet perception and the improved effects of BZYQF, we observed ATP release responses to sucrose solution in a high-glucose (HG) model of taste bud organoids. As shown in Figure 8A, there was no significant difference in ATP release response to DPBS without sucrose among the organoids of the four groups. However, with increasing concentrations of sucrose solutions, the ATP release gradually increased in the organoids of the four groups. However, the organoids in the HG group had significantly reduced ATP release responses to sucrose solutions of different concentrations compared with those in the control group. After treatment for 8 days, the organoids in the BZYQF group had significantly higher ATP release responses to sucrose solutions of different concentrations than those in the HG group. Conversely, this effect of BZYQF on enhancing ATP release responses to sucrose solutions could be inhibited by the Lactisole T1R3 antagonist (Figure 8B–E).

### 2.8. BZYQF Upregulated the mRNA and Protein Expressions of STR Signaling Molecules in the High-Glucose Model of Taste Bud Organoids

**Figure 9:**
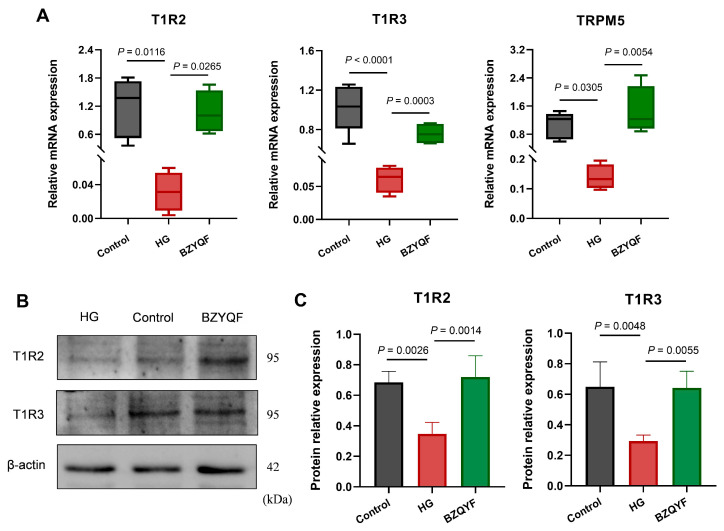
The mRNA and protein expressions of STR signaling molecules in taste bud organoids. The (**A**) mRNA and (**B**,**C**) protein expressions of T1R2 and T1R3 were lower in the organoids of the HG group than those in the control group. BZYQF significantly reversed the reduction in the expressions of STR signaling molecules in the organoids of the HG group. Control group: N = 4~5; HG group: N = 4; BZYQF group: N = 4.

To further investigate the molecular mechanism underlying the sweet taste disorders caused by high glucose concentrations and to better understand the beneficial effects of BZYQF, we measured the mRNA expression of STR signaling molecules in taste bud organoids using RT-qPCR. The results showed that the expression of STR signaling molecules (T1R2, T1R3, and TRPM5) in the organoids was lower in the HG group than in the control group. After treatment for 8 days, BZYQF significantly increased the expression of STRs signaling molecules in the organoids of the HG group (Figure 9A).

We also measured the protein expression of T1R2 and T1R3 in the taste bud organoids using Western blot. The results showed that the T1R2 and T1R3 expression significantly decreased in the organoids of the HG group compared with those of the control group. After treatment for 8 days, T1R2 and T1R3 expression increased in the organoids of the BZYQF group compared with those of the HG group (Figure 9B,C). These results revealed that BZYQF effectively increased the expressions of STR signaling molecules in the organoids of the HG group.

## 3. Discussion

This study demonstrated that, in T2DM rats, BZYQF significantly alleviated T2DM and improved perceptions of sweet and bitter solutions at medium and high concentration, as well as sour solutions at low concentration. In vitro experiments confirmed that BZYQF increased ATP release responses to sucrose solution in a high glucose model of taste bud organoids. Further investigation revealed that BZYQF ameliorated T2DM-induced taste disorders by increasing the number of taste buds and upregulating the expression of STR signaling molecules in the lingual papillae of T2DM rats. The present study provides evidence for the effectiveness of BZYQF in treating T2DM and T2DM-induced taste disorders.

T2DM is one of the most common diseases endangering human health, and the complications caused by long-term hyperglycemia in T2DM patients seriously affect the quality of life and survival status of patients. Among the various complications associated with T2DM, taste disorders are often overlooked. Clinical observations have revealed that T2DM patients have impaired perceptions of taste and particularly, a reduced ability to perceive sweetness. Some studies found that T2DM patients had an increased sweet detection threshold, and that threshold was positively correlated with elevated blood glucose levels [15,16]. This impaired ability to perceive sweetness influences dietary habits and nutrition intake in T2DM patients, further aggravating the diabetic condition. There are many hypoglycemic drugs currently available to treat T2DM, but they have limited effectiveness in treating taste disorders caused by T2DM. Some studies have indicated that drugs such as metformin and glibenclamide might directly contribute to taste impairment in T2DM patients [17]. Therefore, there is an urgent need to find new drugs to treat T2DM and the taste disorders associated with the condition. In traditional Chinese medicine, T2DM belongs to the category of “XiaoKe disease” and it is believed that the impairment of spleen and stomach functions is the main pathogenic cause of T2DM. BZYQF is a classic formula used to improve the functioning of the spleen and stomach [10] and demonstrates significant effectiveness in regulating digestive and immune functions [11]. Pharmacological studies have shown that the active ingredients of several Chinese herbs in BZYQF can alleviate T2DM. Our previous studies demonstrated that astragalus polysaccharides alleviated T2DM in rats by reversing the glucose transporters and STR/GLP-1/GLP-1R signaling pathways in the intestine–pancreatic axis [18]. Additionally, Song et al. found that saikosaponin D reduced insulin resistance and blood glucose levels in T2DM rats by inhibiting the FoxO1/PGC-1α signaling pathway [19]. The present study found that BZYQF significantly improved the symptoms of polyuria, polyphagia, polydipsia, and weight loss in T2DM rats, reduced the levels of FBG, TC, and TG, and increased insulin levels and HOMA-β in the serum of T2DM rats. Furthermore, this study found that there was a significant difference in preference scores and intakes of tastant solution and total solution between the T2DM rats and normal rats in the four tastant tests. This finding suggests that the T2DM rats had disordered perceptions of sweet, bitter, salty, and sour flavors. BZYQF was found to alleviate the impairments in the perception of sweet and bitter solutions at medium and high concentrations as well as sour solutions at low concentration in the T2DM rats. Interestingly, as the concentration of the tastant solutions increased, only the intake of maltose was found to be significantly increased in terms of total solution intake in the experimental rats. Similar results were not found for the other three tastant solutions. These findings reveal that excessive intake of sweet solution aggravated polydipsia in T2DM rats. As is well known, T2DM patients experience typical symptoms such as polydipsia and polyuria, mainly due to hyperglycemia. When T2DM rats consume maltose solutions at high concentrations, it causes hyperglycemia and further exacerbates thirst, resulting in further consumption of maltose solution. Moreover, T2DM rats with a reduced ability to perceive sweetness might consume more maltose solution to achieve the same taste satisfaction, ultimately leading to rapid increases in blood glucose levels and affecting the management of blood glucose stability [6]. These findings reveal that T2DM rats exhibited taste disorders, especially a reduced perception of sweetness, and that BZYQF ameliorated these T2DM-induced taste disorders.

The taste buds are peripheral structures responsible for the taste perception of taste from food and are housed on the lingual papillae. There is a single taste bud in every FP and there are hundreds in CVPs. Previous research found that the density of fungiform papillae containing taste buds is closely related to the intensity of taste perception; subjects with higher taste bud densities gave significantly higher average intensity ratings for sucrose, NaCl, and 6-n-propylthiouracil (PROP, bitterness) [20]. This could be due to the fact that a decrease in the number of taste buds or FPs reduces the binding of tastants with taste receptors, thereby attenuating taste perception. As a risk factor for T2DM, obesity affects taste thresholds and sensitivity in humans. Animal studies have also confirmed that a long-term high-fat diet can reduce the number of taste buds and taste signaling in obese mice, with irreversible effects even after switching to a normal diet [21]. In addition, morphological studies showed that obese individuals exhibited a change in the morphology of taste buds and central taste nuclei, and their FP density was negatively correlated with the degree of obesity [22,23]. A separate study highlighted the severe impairment of taste-related functions and anatomical structures in T2DM patients, characterized by an obvious reduction in FP density (including taste buds) and significant changes in the morphological structure of taste buds [24]. Dhouha et al. and Nesma et al. also observed that T2DM rats had abnormal lingual papillae morphology and reduced numbers of taste buds [25]. Therefore, alterations in lingual papilla morphology and taste bud count among diabetic individuals may increase their taste detection thresholds and change their food preferences, ultimately worsening the progression of diabetes. Consistent with previously reported studies, the present study found that the T2DM rats had a significant reduction in FP densities compared with normal rats, accompanied by loss of taste buds in the FPs. Furthermore, the T2DM rats exhibited an obvious reduction in the numbers of taste buds in the CVPs compared with normal rats, and the morphological structure in the CVPs, especially the furrow, was significantly different between rats in the two groups. After drug treatment, BZYQF significantly improved the numbers of taste buds and morphological structure of FPs and CVPs in T2DM rats. These results suggest that the histopathological changes in the lingual papillae were the cause of the reduced taste perception in T2DM rats, and that BZYQF treatment alleviated this by increasing the number of taste buds and improving the morphological structure of lingual papillae.

The taste buds perceive taste mainly through taste transduction pathways in the taste cells. Type II taste cells are responsible for the recognition of sweet, bitter, and umami flavors through the classical taste transduction of the taste receptor GPCRs/PLCβ2/Ca2+/TRPM5. Different from bitter and umami, the perception of sweetness is predominantly mediated by T1R2 and T1R3 heterodimers. Several studies showed that overall sucrose preference and intake were substantially less in T1R3 knockout mice than those in C57BL/6 (B6) wild-type (WT) mice [26,27]. Notably, obese mice had a reduced response to sweet stimuli, possibly due to a decrease in T1R3 mRNA expression [28]. Genetic studies found that variations in T1R2 and T1R3 were closely linked to the ability to detect sucrose in humans. In particular, the polymorphisms in the promoter region of the T1R3 gene had a notable impact on sucrose perception [29], and the single nucleotide polymorphism (SNP) rs12233832 in the T1R2 gene was found to influence individuals’ perceptions of sweetness and sugar consumption [30]. Similar to T1R2 and T1R3, α-gustducin and TRPM5 are essential for the detection of sugar and sweeteners. Other research found that mice lacking α-gustducin or TRPM5 exhibited diminished responses to bitter, sweet, and umami tastes; α-gustducin knockout mice consumed fewer polycose solutions than WT mice due to impaired taste perception [31]. The TRPM5 knockout mice displayed no preference for saccharin, indicating impaired sweet taste signaling [32]. Evidence from single-cell RNA sequencing of human FPs suggested that expression levels of PLCβ2 and GNAT3 (encoding α-gustducin) were significantly reduced in obese individuals compared with lean individuals [33]. The present study found that mRNA expression of STR signaling molecules of T1R2, T1R3, TRPM5, IP3R, and α-gustducin in the FP and CVP significantly decreased in the T2DM rats compared with normal rats. After drug treatment, BZYQF was able to reverse the mRNA expression of these STR signaling molecules in T2DM rats. Furthermore, the CVP immunofluorescence results further confirmed the mRNA expression levels of T1R2 and T1R3 among the three groups of rats from the protein expression levels. In addition, the present study found that most of the compounds in BZYQF had good binding activity to T1R2, T1R3, or heterodimers of T1R2/T1R3; although the content of these compounds such as flavonoids was not high, there were many of these compounds and their binding sites with STRs were consistent. Therefore, we speculate that various compounds in BZYQF, especially flavonoids, might exert a synergistic effect on regulating STRs and improving taste disorders in T2DM rats. These results suggest that reduced expressions of STR signaling molecules in the FPs and CVPs were another cause of impaired taste perception in T2DM rats, while BZYQF ameliorated taste disorders caused by T2DM by upregulating the expression of STR signaling molecules.

Taste bud organoids are important tools for studying taste perception and related disorders and are of great importance for screening drugs to improve taste disorders. Ren et al., through a calcium-imaging experiment, found that a subset of cells in taste bud organoids could detect sweet, bitter, and salty substances, and the organoids’ response to Benzethonium could be hindered by the PLCβ2 blocker U73122 [34]. In another study, taste bud organoids were placed on a 64-channel microelectrode array (MEA) chip to monitor the electrophysiological signals generated by various taste stimuli [35]. Furthermore, taste bud organoids derived from a mouse model of oral mucositis were used to evaluate the therapeutic effect of drugs on radiation-induced taste loss [36]. Considering the potential of taste bud organoids in simulating and treating diabetes-related taste disorders, the present study successfully cultured taste bud organoids that expressed sweet taste receptors of T1R2 and T1R3 and responded to sucrose solution in vitro. By measuring sucrose-evoked ATP release, the present study revealed that the taste bud organoids in the HG group showed significantly more attenuated ATP release responses to sucrose solutions compared with those in the control group. However, BZYQF treatment significantly increased ATP release responses to sucrose solutions in the organoids in the HG group. Interestingly, this effect was significantly inhibited by the T1R3 antagonist lactisole. Further mechanistic studies showed that the mRNA and protein expression levels of STR signaling molecules in the taste bud organoids of the HG group were significantly lower than those in the control group, and BZYQF treatment effectively increased the expression levels of STR signaling molecules in the taste bud organoids of the HG group. These results suggest that a high-glucose environment induces taste disorders, and that BZYQF treatment can ameliorate HG-induced taste disorders by activating STR signaling pathways.

## 4. Materials and Methods

### 4.1. Preparation of BZYQF

The BZYQF consisted of 10 Chinese herbs: *Astragalus membranaceus* (Fisch.) Bunge (honey roasted, HuangQi, 200 g), *Glycyrhiza uralensis* Fisch. (honey roasted, GanCao, 100 g), *Codonopsis pilosula* (Franch.) Nannf. (DangShen, 60 g), *Atractylodes macrocephala* Koidz. (stir fried, BaiZhu, 60 g), *Angelica sinensis* [Oliv.] Diels. (DangGui, 60 g), *Citrus reticulata* Blanco. (ChenPi, 60 g), *Bupleurum chinense* DC. (ChaiHu, 60g), *Cimicifuga heracleifolia* Kom (ShengMa, 60 g), *Ziziphus jujuba* Mill (DaZao, 40 g), and *Zingiber officinale* Roscoe. (ShengJiang, 20 g). In accordance with the Ministry of Health for Traditional Chinese Medicine Formulations (Volume 7), BZYQF was manufactured into BuZhong YiQi Pill by JiuZhiTang Co., Ltd. (National Medical Approval No. Z43020143, Changsha, China). The manufacturing method was briefly introduced as follows [37]: ShengJiang and ChenPi were firstly taken to extract the volatile oil. Their remaining residues were boiled in water with HuangQi, BaiZhu, ShengMa, ChaiHu, DaZao, and one half of the GanCao, to obtain the decoction by filtration. The decoction was concentrated into a thick paste, while DangShen, DangGui, and the other half of the GanCao were crushed into fine powder. Finally, all these volatile oils, thick pastes, and powders were mixed to manufacture pills of BZYQF. According to the drug instructions, 8 pills were equivalent to 3 g of raw herbs.

The pills of BZYQF were heated with distilled water at 100 °C to produce a water decoction with a concentration of 1 g/mL. For the in vitro experiment, the water decoction was centrifuged at 12,000× *g* for 10 min to remove insoluble components and the supernatant was passed through 0.22 mm filters sequentially for sterilization.

### 4.2. Characterization of Composition of BZYQF

Compositional characterization of BZYQF was conducted using widely targeted metabolomics techniques based on UPLC-MS/MS, according to our previous method [37]. The UPLC analytical conditions were as follows. The column was an Agilent SB-C18 (1.8 µm, 2.1 mm × 100 mm); the injection volume was 2 μL; the flow velocity was adjusted to 0.35 mL/min; the column oven was set at 40 °C. The mobile phase consisted of solvent A (pure water with 0.1% formic acid) and solvent B (acetonitrile with 0.1% formic acid). Sample measurements were performed using a gradient program as follows. Initial conditions of 95% A, 5% B were used. A linear gradient was then programmed to 5% A, 95% B within 9 min and a composition of 5% A, 95% B was maintained for 1 min. Subsequently, a composition of 95% A, 5.0% B was set within 1.1 min and maintained for 2.9 min. The effluent was alternatively connected to a QTRAP-MS. The operating parameters of the ESI source were as follows: 500 °C source temperature; 5500 V (positive ion mode)/−4500 V (negative ion mode) ion spray voltage. Curtain gas and ion source gas I and II were set at 25, 50, and 60 psi, respectively. Collision gas (nitrogen) was set to medium and collision-activated dissociation was set to high. The multiple reaction monitoring (MRM) model was used to acquire triple quadrupole MS scans. Specific MRM ion pairs were monitored according to the eluted metabolites in each period. Based on a self-created database (Metware Biotechnology Inc., Wuhan, China), the secondary spectral information was used to determine metabolites.

For the purpose of quality control, the content of calycosin-7-glucoside, a particularly abundant flavonoid, was determined via HPLC (#UltiMate3000, Thermo Scientific, Waltham, MA, USA) [37]. In addition, the best-known active components of BZYQF, including total contents of carbohydrates, reducing sugar, polysaccharides, flavonoids, polyphenol, and saponin, were determined by a colorimetric method [38,39].

The present study identified 1909 compounds in BZYQF via UPLC-MS/MS. The primary classifications of these compounds, arranged in descending order of amount, were flavone (24.46%), phenolic acids (13.31%), amino acids and their derivatives (8.64%), alkaloids (7.81%), lipids (7.39%), terpenoids (6.91%), organic acids (6.71%), lignans and coumarins (6.44%), and other categories (28.33%). Among these, flavone was present in the highest amount, with 467 secondary flavonoids. These flavonoids mainly included flavone (32.55%), xanthone alcohol (17.34%), isoflavone (13.49%), dihydroflavonoid (11.13%), chalcone (8.99%), and other flavonoids (16.48%) (as shown in the Supplementary Materials). In addition, the calycosin-7-glucoside content was 0.83 mg/g pills (as shown in the supplementary data). The contents of total carbohydrates, reducing sugar, and polysaccharides were 146.72 ± 2.59 mg/g, 82.22 ± 1.11 mg/g, and 59.78 ± 2.86 mg/g, respectively. The contents of total flavonoids, total polyphenol, and total saponin were 23.45 ± 4.39 mg/g, 123.79 ± 2.68 mg/g, and 5.32 ± 0.04 mg/g, respectively. A detailed report can be found in our previous study [37].

### 4.3. Molecular Docking of BZYQF Compounds with STRs

Molecular docking was utilized to speculate on the potential compounds in BZYQF that targeted STRs and evaluate their binding affinity with STRs. The 2D structures of all the compounds identified in the BZYQF were obtained from the PubChem database (https://pubchem.ncbi.nlm.nih.gov/, accessed on 11 May 2025) and stored in SDF format. Preprocessing of these ligands was performed using OpenBabel 3.1.1, including correction of the protonation state at pH = 7.4, calculation of Gasteiger charges, and detection of rotatable bonds. The 3D structures of STRs (9NOR: human STRs composed of T1R2 and T1R3) were obtained from the RCSB Protein Data Bank (PDB) database (http://www.rcsb.org, accessed on 11 May 2025) and stored in PDB format. Preprocessing of STRs were performed using AutoDock 4.2.6, including removal of water, solvent molecules and other non-protein structures, removal of non-standard amino acids and lone pairs of electrons, addition of hydrogens, determination of a single conformation for residues with multiple conformations, and calculation of Gasteiger charges. Files for all preprocessed proteins and ligands were recorded using the PDBQT format. Molecular docking was performed using AutoDock-Vina software (version 1.1.2), where the minimum binding affinity was the criterion for conformational stability analysis. Affinity (kcal/mol) indicates the ability of the protein-compound binding, where a lower value reflects a stronger binding of compound to protein. Less than −5 Kcal/mol or −7 Kcal/mol indicated a good or strong binding activity between the compound and the protein, respectively. Discovery Studio 2019 software was used to visualize the docking results.

### 4.4. Induction of T2DM Rats and BZYQF Administration

Male Sprague–Dawley (SD) rats with a body weight of 190–210 g were provided by the Laboratory Animal Center of Southern Medical University, and the production license number was SCXK (Guangdong) 2021-0041. SD rats were bred in the Experimental Animal Center of Guangdong Pharmaceutical University at a room temperature of 22–25 °C and a relative humidity of 60–70% with a 12/12 h light/dark cycle. T2DM induction was performed according to our previous method [40,41,42]. A brief description follows. After acclimation for 3 days, SD rats were randomly divided into two groups of normal (N = 8) and model (N = 22) animals. During the entire experiment, the rats in the normal group were fed a basal diet (nutritional composition: 24% protein, 61% carbohydrate, 5% fat, 4% cellulose, 5% mineral mix S10026B, and 1% vitamin mix AIN-76A; the energy value was 3.85 kcal/g and fat energy accounted for 12% of total energy), while the rats in the model group were fed with a high-fat diet (nutritional composition: 24% protein, 41% carbohydrate, 24% fat, 5% cellulose, 5% mineral mix S10026B, and 1% vitamin mix AIN-76A. The energy value was 4.76 kcal/g and fat energy accounted for 45% of total energy). Rat feed was manufactured by Guangdong Medical Laboratory Animal Center and the energy value (kcal/g) was calculated according to the following formula: [mass percent of protein (g/kg) *×* 4 kcal/g + mass percent of carbohydrate (g/kg) × 4 kcal/g + mass percent of fat (g/kg) × 9 kcal/g]/1000]. After feeding for 8 weeks, rats in the model group received a single intraperitoneal injection of 30 mg/kg streptozotocin (STZ, #V900890, Sigma-Aldrich, St. Louis, MI, USA) freshly dissolved in 0.1 mol/L sodium citrate buffer with a pH 4.5, while rats in the normal group were injected with an equivalent volume of citrate buffer after fasting for 12 h. One week after STZ injection, caudal venous blood was collected from experimental rats to measure fasting blood glucose (FBG) with a glucometer (#HGM-114, OMROM, Dalian, China). The T2DM rat model was successfully induced when rats in the model group achieved FBG > 11.1 mmol/L, with accompanying symptoms of polydipsia, polyphagia, polyuria, and weight loss. Subsequently, the 22 STZ-induced model rats were randomly divided into three groups, i.e., BZYQF (N = 8), T2DM (N = 8), and metformin (N = 6). Based on our initial experimental findings for three dosages of 4, 8, and 12 g/kg/d BZYQF, and previous research [43], the rats in the BZYQF group received intragastric administration of 8 g/kg/d BZYQF. According to clinical administration principles, the rats in the metformin group received intragastric administration of 210 mg/kg/d metformin. In order to eliminate the potential impact of the administration volume on the experimental results, the rats in the T2DM group and the normal group were intragastrically administered physiological saline at about 3.0 mL/kg/d, which was calculated based on the body weight of the rats and an administration dosage of 8 g/kg/d. All rats received intragastric administration for 8 weeks. During the experiment, these indicators of body weight, food intake, and water intake were determined once weekly in the experimental rats. The present study strictly complied with animal welfare ethics and protection regulations and was approved by the Laboratory Animal Ethics Committee of Guangdong Pharmaceutical University (approval number gdpulacspf2022203).

### 4.5. Two-Bottle Preference Test

The two-bottle preference test was utilized to evaluate the sensitivity of experimental rats to different tastant solutions. This test was performed as previously described [44]. Briefly, rats in three groups were housed in separate cages. Each rat was given two identical bottles of distilled water and trained to drink from the bottles. After training for one week, the rats were allowed to drink freely from two bottles for 48 h, one bottle containing distilled water and the other containing a tastant solution. After 24 h, the volume of solution consumed in the two bottles was replenished and the bottles’ positions (right or left) were changed to avoid volume and position effects. Rats were provided with tastant solution at three different concentrations from the lowest to the highest concentration. The tastant solutions used for the two-bottle preference test included sweet (5, 10, and 50 mM of maltose (#M874782, Macklin, Shanghai, China)), bitter (0.01, 0.03, and 0.1 mM of quinine hydrochloride (#Q107527, Aladdin, Shanghai, China)), salt (75, 150, and 300 mM of NaCl (#S805275, Macklin)); and sour (1, 2.5, and 5 mM of citric acid (#C805019, Macklin)). Between tests with the two tastant solutions, the rats were given regular drinking water again for two days before ingesting the next tastant solutions. The intakes of total solution and tastant solution were recorded in each tastant test at 24 and 48 h. The preference score for the tastants was calculated as follows: tastant solution intake/total solution intake (tastant solution intake + distilled water intake). According to this calculation formula, a preference score between 0.5 and 1 indicated that rats preferred the tastant solution over water, while a preference score between 0 and 0.5 indicated that rats avoided the tastant solution.

### 4.6. Sample Collection and Processing

When the experiment concluded, experimental rats were anaesthetized with 30 mg/kg of sodium pentobarbital (Beijing Chemical Reagent Company, Beijing, China) by intraperitoneal injection after fasting for 12 h. These rats were then sacrificed by draining blood from the abdominal aorta. Each rat’s tongue was removed from its intraoral root attachment at the level of the trachea. Tongue tissues were sectioned transversely and posteriorly to the intermolar eminence and divided into apical and laryngeal segments. After washing with PBS, these tissue samples were quickly frozen in liquid nitrogen and then stored in a freezer at −80 °C. Part of each tongue tissue sample was fixed with 4% paraformaldehyde in PBS at 4 °C. The collected blood samples were placed at 4 °C for 2 h and centrifuged at 3000× *g* for 15 min to collect serum. These serum samples were stored in a freezer at −80 °C.

### 4.7. Serum Biochemical Determination

The serum levels of FBG, total cholesterol (TC), and triglyceride (TG) were measured using commercial test kits (Shenzhen Mindray Biomedical Electronics Co., Ltd., Shenzhen, China) on a biochemical analyzer (BS-180, Mindray, Shenzhen, China). Fasting insulin levels were assayed on a microplate reader (Elx-800, BioTek, Winooski, Vermont, USA) using a rat insulin ELISA kit (#ER1113, FineTest Biotech, Wuhan, China). The experiment was carried out according to the manufacturer’s protocols and instructions.

### 4.8. Counting of Fungiform Papillae

The apical tongue segments containing FP were stained with 1% methylene blue tetrahydrate (#C12027752, Macklin) for 5 min [45]. The FPs on the right and left sides of the midline sulcus were counted from the median eminence to the apex using a dissecting stereo-microscope (#SZX7, Olympus, Tokyo, Japan) equipped with a digital camera (EOS M6, Cannon, Tokyo, Japan).

### 4.9. Hematoxylin and Eosin Staining and Structural Measurement of the FP and CVP

Morphological and dimensional assessments of FP and CVP, including taste buds, were carried out using hematoxylin and eosin (HE) staining on the tongue specimens of rats from the three groups. According to a standard protocol, the apical and laryngeal segments of the tongue were separately embedded in paraffin. These embedded specimens were successively cut into 4–5 µm slices via a microtome. These sliced specimens were then deparaffinized with xylene and rehydrated in a graded ethanol sequence at decreasing concentrations. Finally, these specimens were stained with HE and coverslipped with a mounting reagent (neutral balsam, #G8590, Solarbio, Beijing, China). Images were captured using a microscope (#EVOS M5000, Thermo Scientific) and quantitative analysis was performed using ImageJ v1.8.0 software (NIH, Bethesda, MD, USA). The numbers of taste buds in the CVPs were counted, the width (the distance between two taste buds directly opposite each other) and the depth (the distance between the upper entrance and the lower end of a papilla furrow) of the furrow was measured, and the average taste bud area and the width of the CVP (the separation between the two furrows at their anteroposterior midpoint) were determined [46]. All the above parameters were measured 4–6 times and measurements were repeated 3 times independently, with the average value used for statistical analysis. HE staining for taste bud organoids was also performed using this method.

### 4.10. Immunofluorescence

The 4–5 µm thick CVP slides were obtained from the tongue specimens of the rats in three groups using the HE staining method. Slides were subjected to antigen repair in citric acid buffer (pH 6.0) for 20 min at 90 °C. Blocking was carried out with 0.1% TritonX-100 and 5% bovine serum albumin in PBS for 30 min. The slides were incubated with primary antibodies against GNAT3 (1:200, #bs-6149R, Bioss, Beijing, China), T1R2 (1:500, #bs-9599R, Bioss), and T1R3 (1:500, #NB100-98792, Novus Biologicals, Littleton, Colorado, USA) overnight at 4 °C. The next day, the slides were washed and incubated sequentially with a secondary antibody (Dylight 488-conjugated goat anti-rabbit antibody, 1:1000, #ab150077, Abcam, Cambridge, UK; Dylight 550-conjugated goat anti-rabbit antibody, 1:1000, #BA1135, BOSTER, Wuhan, China) and counterstained with DAPI to visualize the nuclei. As a negative control, immunofluorescence staining without primary antibodies was used. Immunostaining for taste bud organoids was performed using whole-mount staining as previously described [47]. Organoids re-suspended in Matrigel were fixed with 4% paraformaldehyde for 30 min. The remaining steps were consistent with the immunostaining for tissue slices. The images were examined using a fluorescence image system (#EVOS M5000, Thermo Scientific).

### 4.11. Taste Bud Organoid Cultures

Taste bud organoids were prepared from the CVPs as previously described [14]. Briefly, the tongues from euthanized rats were isolated, and an enzyme mixture solution consisting of dispase (2 mg/mL, #Z8030, Solarbio) and collagenase (1 mg/mL, #C6885, Sigma-Aldrich) in PBS was injected under the epithelium. After incubation for 30 min at 37 °C, the tongue epithelium was carefully peeled away from the underlying muscle tissue. The epithelium of CVP was dissected and incubated with TrypLE Expless (#12604-021, Gibco, Carlsbad, CA, USA) for 30 min at 37 °C. After centrifugation at 1000× *g* for 5 min, the cell pellet was resuspended in DMEM with 50% Matrigel (#356234, Corning, NY, USA) and seeded into prewarmed 24-well culture plates. After Matrigel polymerization for 10 min at 37 °C, taste bud organoid culture medium based on DMEM/F12 medium (Gibco) supplemented with Wnt3a (100 ng/mL, PeproTech, Cranbury, NJ, USA), R-spondin-1 (200 ng/mL, R&D systems, Minneapolis, MN, USA), Noggin (100 ng/mL, PeproTech), Y27632 (10 μM), N-acetylcysteine (1 mM, Sigma), epidermal growth factor (50 ng/mL, PeproTech), N2 (1%, Gibco), and B27 (2%, Gibco) was added to the plate and replaced every 3 days. For passage culture, taste bud organoids were incubated with TrypLE solution for 30 min at 37 °C and then dissociated into small pieces. Single cells were collected through cell strainer and pelleted by centrifuging at 700× *g* for 5 min. The cell pellet was re-embedded into fresh Matrigel and plated in 24-well plates. All experiments including live imaging, HE staining, immunostaining, organoid modelling, ATP release, PCR, and Western blotting were conducted using organoids after 2–4 passages.

### 4.12. Organoid-Forming Efficiency Assays and Live Imaging

According to the passage culture method, organoids were dissociated into single cells and these single cells were replated at a density of 1000 cells on a 96-well plate previously coated with Matrigel. Each well was supplemented daily with 10 μL of Cell Counting Kit-8 (CCK8, #C0038, Beyotime, Shanghai, China) and incubated for 1 h at 37 °C. The absorbance at 450 nm was measured on the microplate reader (#SynergyTM H1, Biotex, Houston, TX, USA) to quantify the numbers of organoids. Organoid-forming efficiency was determined by quantifying the organoids’ numbers and size under a bright-field microscope. The growth curves were plotted using GraphPad Prism (version 9.4.0) based on the absorbance values obtained from the CCK8 assays. Imaging was performed in organoid culture medium under 5% CO_2_/37 °C incubation (BPN-80CH, Shanghai bluepard instruments Co., Ltd., Shanghai, China), via an inverted microscope (Olympus BX51).

### 4.13. High-Glucose Model of Organoids and Measurement of Sucrose-Induced ATP Release

The high-glucose model was constructed according to our preliminary experimental results and a previous study [48]. An equal number of taste bud organoids were seeded onto 96-well plates and cultured in DMEM/F12 basal medium with 17.5 mM glucose, which was selected as the control glucose concentration. Glucose (#D992007, Macklin) was added to the basal medium at a concentration of 30 mM to create a high-glucose environment. After an initial 4-day culture period, taste bud organoids were exposed to medium containing control glucose (17.5 mM, control group), high glucose (30 mM, HG group), BZYQF (30 mM glucose and 10 mg/mL BZYQF, BZYQF group), or BZYQF + lactisole (10 μM lactisole added to BZYQF, Lactisole group; #GC44024, GLPBIO, Montclair, California, USA) for an additional 6 days. The basal medium was renewed every day and the concentrations of glucose, BZYQF and lactisole in the medium were kept constant in four groups. After continuous culture for 6 days, the organoids’ ATP release response to sucrose solution at different concentrations was measured using luciferase, as described in a previous report [49]. Briefly, after washing three times with PBS, organoids were stimulated with 100 μL sucrose solution at different concentrations (0, 5, 10, 20, and 30 mM sucrose solutions prepared with DPBS) for 30 sec, respectively. Within 1 min after the end of stimulation, the stimulated solution was collected and mixed with an equal volume of luciferase reagent (Enhanced ATP Assay Kit, #S0027, Beyotime). The mixed solution was transferred to a black 96-well plate for luminescence measurement. In the presence of ATP, luciferin was oxidized by luciferase, resulting in the production of light proportional to the amount of ATP. Readings were recorded in arbitrary relative light units (RLU) using the software provided with the Multimode Microplate Reader (Varioskan™ LUX, Thermo Scientific).

### 4.14. RNA Isolation and Real-Time Quantitative PCR

Total RNA was extracted from CVP and FP tissues and organoids using Trizol (#9109, TaKaRa, Kusatsu, Japan). The concentration and purity of total RNA were determined using a microvolume spectrophotometer (NanoDrop Lite, Thermo Scientific). Total RNA with a 260/280 value of 1.9–2.1 was reverse transcribed into cDNA on a T100 Thermal Cycler (Bio-Rad, Hercules, CA, USA) using FastKing gDNA Dispelling RT SuperMix (#Y1815, Tiangen Biotech, Beijing, China). The RT thermal cycle was 42 °C for 15 min and 95 °C for 3 min. Real-time quantitative PCR (RT-qPCR) was performed using a CFX Connect Real-Time System (Bio-Rad) with 2 × Color SYBR Green qPCR Master Mix (#A0012-R2, EZBioscience, Roseville, MN, USA) and gene-specific primers (Table 2). The PCR thermal cycle was run at an initial denaturation of 95 °C for 5 min, followed by 40 repeated cycles of 95 °C for 10 sec and 60 °C for 30 sec. Relative expression of the genes of interest was quantified using the 2^−ΔΔCt^ method, with β-actin as the reference gene. The PCR experiment was repeated at least three times for each gene. All operations were carried out according to the manufacturer’s instructions.

### 4.15. Western Blot

Taste bud organoids were homogenized for 30 min on ice using RIPA lysis buffer with 1 mM PMSF (#P0013B, Beyotime; #P0100, Solarbio). Total protein content was quantified using a NanoDrop Lite microvolume spectrophotometer (Thermo Scientific). For this, 40 μg of total protein was prepared using Sample Loading Buffer (Biosharp biochemistry Co., Ltd., Hefei, China) and heated to 100 °C for 5 min. These proteins were separated by SDS-PAGE and transferred onto polyvinylidene fluoride membrane (Mini-Protean Tetra Cell and Mini Trans-Blot Electrophoretic Transfer Cell, Bio-Rad, USA). The membrane was blocked for 1 h at room temperature in blocking buffer (PBS, 0.1% tween-20) with 5% skimmed milk. The proteins in the membrane were then incubated overnight at 4 °C with primary antibodies against T1R2 (1:1000, bs-9599R, Bioss), T1R3 (1:500, NB100-98792, Novus Biologicals), and β-actin (1:5000, #R23613, Zen-BioScience, Chengdu, China). After washing in phosphate-buffered saline with Tween 20 (PBST) three times, these proteins were incubated for 2 h at room temperature with secondary antibodies (1:6000, #BS13278, Bioworld Technology, Nanjing, China). The protein blots were stained with ECL luminous solution after washing with PBST three times. Chemiluminescence signals were collected using GeneGnome XQR gel imaging systems (Syngene, Cambridge, UK) and quantified using ImageJ v1.8.0 software (National Institutes of Health, Bethesda, MD, USA).

### 4.16. Statistical Analysis

GraphPad Prism (version 9.4.0) was used for data analysis and generation of the figures. One-way ANOVA was used to compare the difference among the multiple groups and Student’s unpaired *t*-test was used to compare the means of the observation parameters between the two groups. A value of *p* < 0.05 was considered statistically significant. The results are expressed as mean ± standard deviation. All experiments were performed at least three times.

## 5. Conclusions

**Figure 10:**
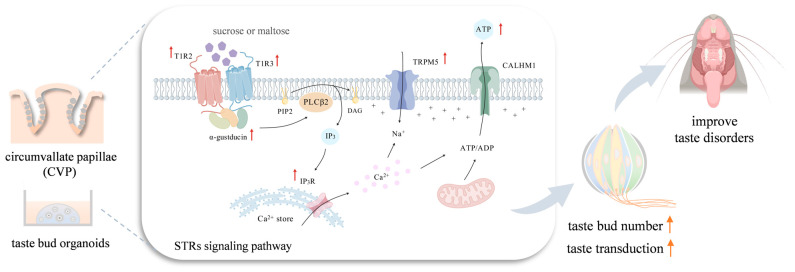
The molecular mechanism of BZYQF ameliorating taste disorders caused by T2DM. BZYQF was able to increase (↑) the numbers of taste buds and expression levels of STR signaling molecules (T1R2, T1R3, α-gustducin, IP3R and TRPM5) in the FP and CVP of T2DM rats. The figure was created with BioGDP.com.

The present study found that BZYQF not only improved the symptoms and the metabolism of glucose and lipids in T2DM rats but also improved impaired perceptions of sweet, bitter, and sour tastant solutions in T2DM rats, especially sweet. In vitro experiments confirmed that a high-glucose environment reduced ATP release responses to sucrose solution in taste bud organoids, while BZYQF reversed organoid disorders induced by glucose at high concentration. Further research into the mechanism revealed that BZYQF ameliorated T2DM-induced taste disorders by increasing the numbers of taste buds and the expression of STR signaling molecules in the FPs and CVPs of T2DM rats (Figure 10). Molecular docking revealed that various compounds in BZYQF, especially flavonoids, might exert a synergistic effect on regulating STRs and improving taste disorders in T2DM rats. The present study confirmed that BZYQF was able to alleviate T2DM and T2DM-induced taste disorders and explored its possible molecular mechanism. However, this study has some shortcomings. For example, this study failed to conduct an in-depth study on bitter and sour, and the reasons for reduced numbers of taste buds caused by T2DM also need to be further investigated. In addition, this study predicted some potential compounds from BZYQF that may regulate STRs, and the activities of these compounds still need to be confirmed in vitro and in vivo. Considering the differences in taste systems between rodents and humans, the findings of this study should be confirmed through clinical trials in humans.

## Figures and Tables

**Table 1:** Biochemical indicators in serum of experimental rats.

Group	Normal	T2DM	BZYQF	Metformin
FBG (mmol/L)	6.22 ± 0.95	21.59 ± 3.06 ^###^	16.43 ± 2.14 **	15.66 ± 4.22 **
Insulin (μU/mL)	13.43 ± 2.08	5.06 ± 0.86 ^###^	10.05 ± 3.33 **	/
HOMA-β	86.86 ± 9.80	5.67 ± 1.01 ^###^	15.56 ± 4.10 *	/
TC (mmol/L)	2.04 ± 0.36	5.75 ± 1.68 ^###^	2.61 ± 0.61 ***	1.96 ± 0.73 ***
TG (mmol/L)	0.61 ± 0.27	12.39 ± 4.10 ^###^	1.83 ± 0.99 ***	1.05 ± 0.59 ***

Compared with normal group, ^###^ *p* < 0.001. Compared with T2DM group, * *p* < 0.05, ** *p* < 0.01, *** *p* < 0.001. The number of rats in each group was the same as in Figure 2.

**Table 2:** The product sizes and sequences of primers used in RT-qPCR.

Gene	Sequences (5′-3′)	Product Sizes (bp)
β-actin	Forward: GGAGATTACTGCCCTGGCTCCTA	103
	Reverse: GACTCATCGTACTCCTGCTTGCTG	
T1R2	Forward: TTCTCATGCTTCTGCCGACAG	196
	Reverse: GCCAATCTTGAAGACACACACGA	
T1R3	Forward: AACAACCAATGGCTCACCTCC	156
	Reverse: AAAGCCATCAAGTACCAGGCAC	
TRPM5	Forward: TCCGCCGTGTGCTCTACAGG	159
	Reverse: GCAGGAGAATGACCAGCCAGTTG	
IP3R	Forward: CCGTACAAGTACGTGCTGCGTCTC	243
	Reverse: GCCGATCTGAGACTGCATGACGC	
α-gustducin	Forward: ACAGTAACACGTTGCAGTCCATCC	146
	Reverse: CTGAGGCGTCATGTCACCATCTTC	

## Data Availability

The original contributions presented in this study are included in the article/Supplementary Materials. Further inquiries can be directed to the corresponding author.

## Supplementary Materials

The following supporting information can be downloaded at: https://www.mdpi.com/article/10.3390/ph18060838/s1, Figure S1: MRM detection of multimodal maps-N; Figure S2: MRM detection of multimodal maps-P; Figure S3: BZYQF HPLC maps; Figure S4: calycosin glucoside HPLC maps; Table S1: All compounds were identified in BZYQF by UPLC-MSMS.

## Abbreviations

The following abbreviations are used in this manuscript:

T2DM	type 2 diabetes mellitus
BZYQF	Buzhong yiqi formula
STRs	sweet taste receptors
FBG	fasting blood glucose
TC	total cholesterol
TG	triglyceride
FP	fungiform papillae
CVP	circumvallate papillae
T1R2/3	taste receptor, type 1, member 2/3
IP3R	inositol 1,4,5-trisphosphate receptor type 1
TRPM5	transient receptor potential cation channel subfamily M member 5
PLCβ2	phospholipase C beta 2
ATP	adenosine triphosphate
