# Supplementary figures and images for: BuZhong YiQi Formula Alleviates Taste Disorders in Rats with Type 2 Diabetes Mellitus by Increasing the Number of Taste Buds and the Expression of Signaling Molecules in Taste Transduction Pathways
Source: Pharmaceuticals (Basel). 2025 Jun 3;18(6):838. doi: 10.3390/ph18060838 (PMC12196184; doi:10.3390/ph18060838)

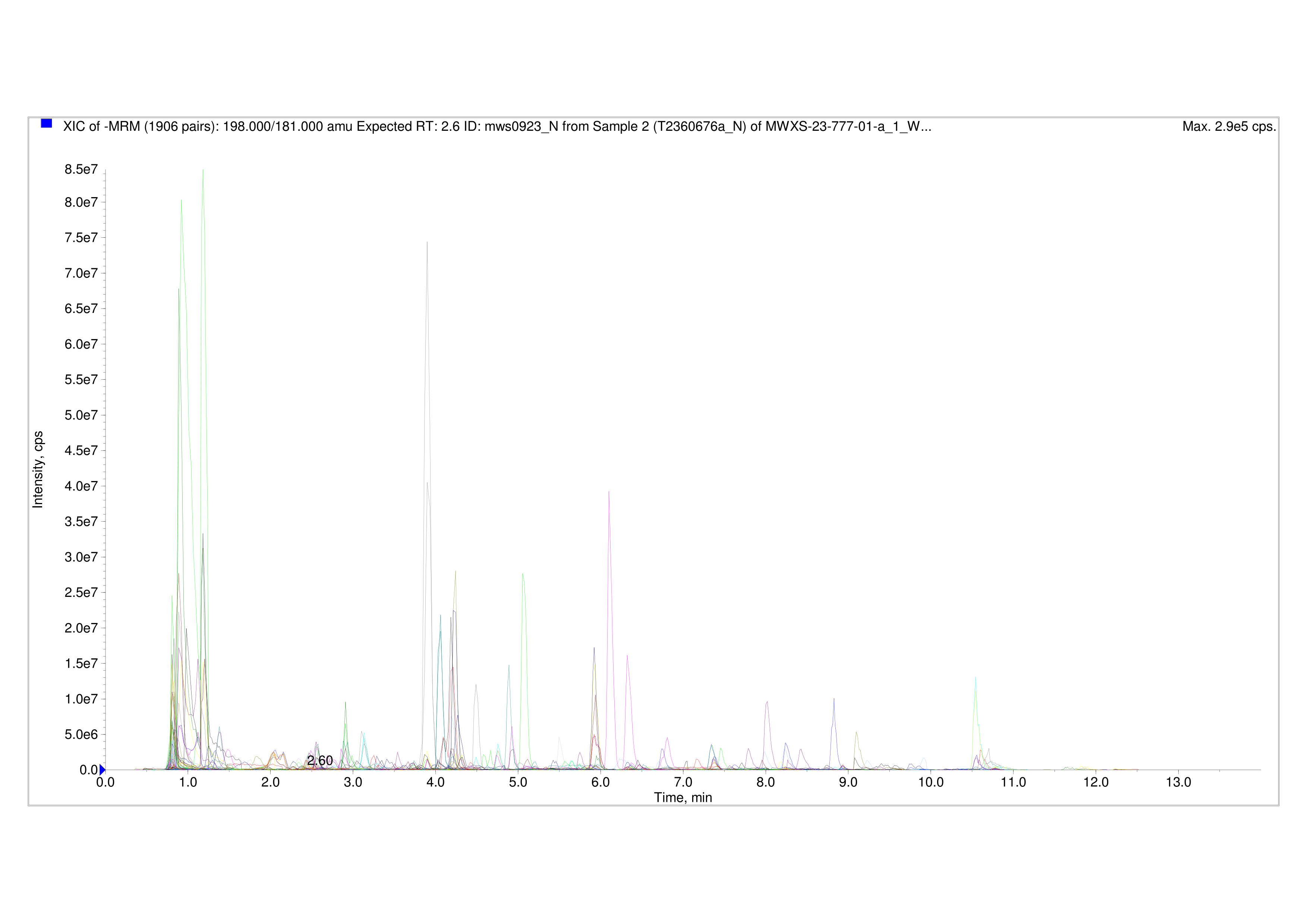

Supplement: Supplementary file 1 [file pharmaceuticals-18-00838-s001.zip › Figure S1 MRM_detection_of_multimodal_maps-N.tif]

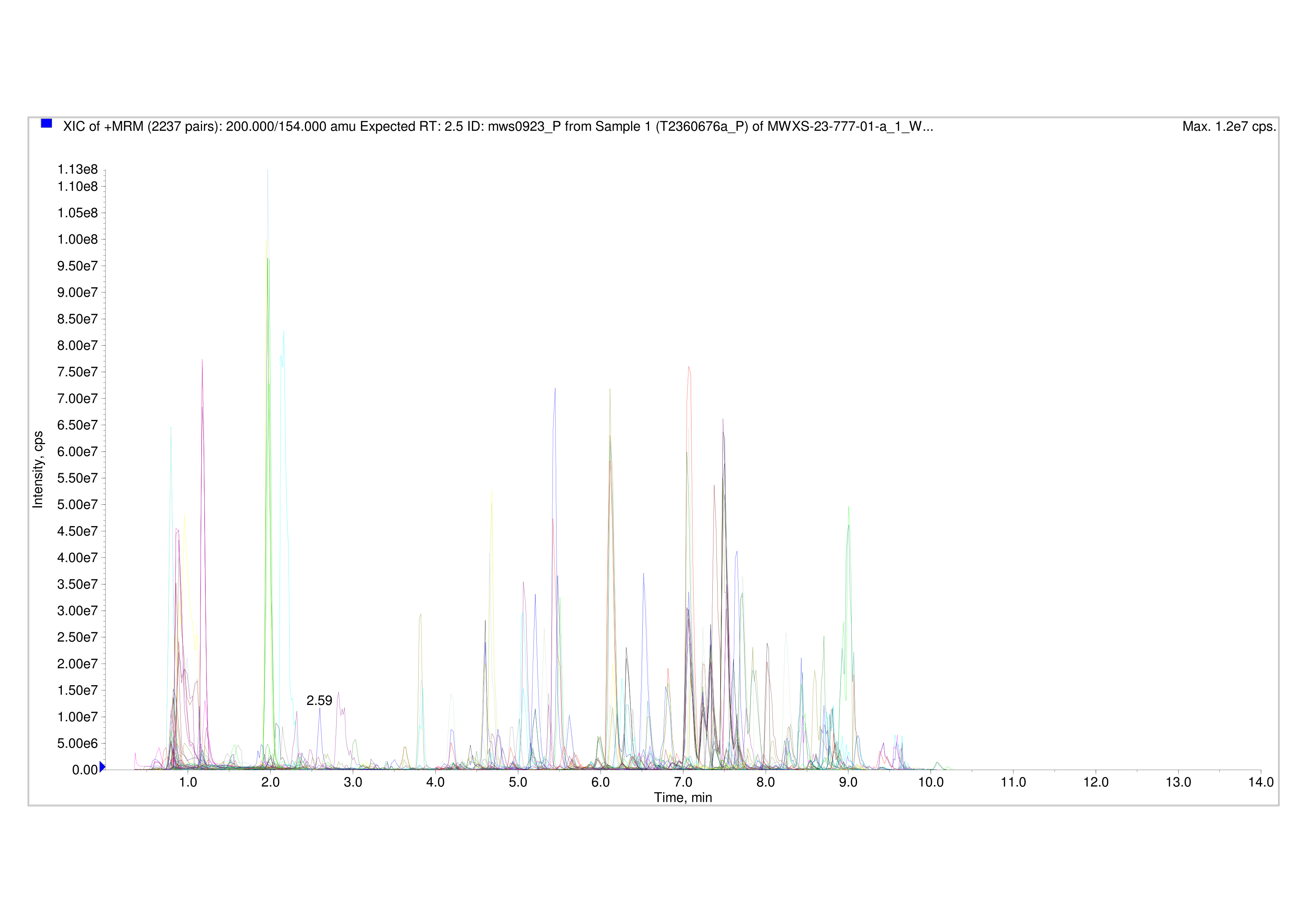

Supplement: Supplementary file 1 [file pharmaceuticals-18-00838-s001.zip › Figure S2 MRM_detection_of_multimodal_maps-P.tif]

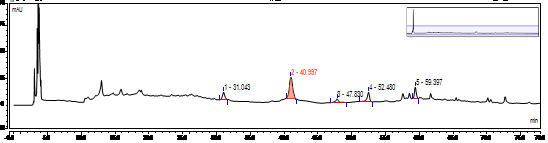

Supplement: Supplementary file 1 [file pharmaceuticals-18-00838-s001.zip › Figure S3 BZYQF_HPLC_maps.png]

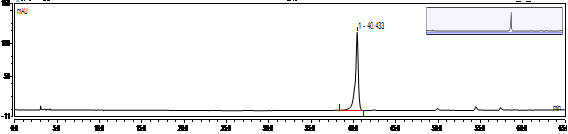

Supplement: Supplementary file 1 [file pharmaceuticals-18-00838-s001.zip › Figure S4 calycosin_glucoside_HPLC_maps.png]
